# Rapid Prototyping Technologies: 3D Printing Applied in Medicine

**DOI:** 10.3390/pharmaceutics15082169

**Published:** 2023-08-21

**Authors:** Małgorzata Oleksy, Klaudia Dynarowicz, David Aebisher

**Affiliations:** 1 Students English Division Science Club, Medical College of the University of Rzeszów, University of Rzeszów, 35-959 Rzeszów, Poland; mo121935@stud.ur.edu.pl; 2 Center for Innovative Research in Medical and Natural Sciences, Medical College of the University of Rzeszów, University of Rzeszów, 35-310 Rzeszów, Poland; kdynarowicz@ur.edu.pl; 3 Department of Photomedicine and Physical Chemistry, Medical College of the University of Rzeszów, University of Rzeszów, 35-959 Rzeszów, Poland

**Keywords:** 3D printing, applications of biomaterials in medicine, polymer biomaterials

## Abstract

Three-dimensional printing technology has been used for more than three decades in many industries, including the automotive and aerospace industries. So far, the use of this technology in medicine has been limited only to 3D printing of anatomical models for educational and training purposes, which is due to the insufficient functional properties of the materials used in the process. Only recent advances in the development of innovative materials have resulted in the flourishing of the use of 3D printing in medicine and pharmacy. Currently, additive manufacturing technology is widely used in clinical fields. Rapid development can be observed in the design of implants and prostheses, the creation of biomedical models tailored to the needs of the patient and the bioprinting of tissues and living scaffolds for regenerative medicine. The purpose of this review is to characterize the most popular 3D printing techniques.

## 1. Introduction

Conventional bone scaffold manufacturing techniques, such as phase inversion, solvent casting, leaching or electrospinning, are often unable to take into account the fine architectural details of the manufactured elements, control product porosity or ensure high reproducibility in production [1,2]. The use of 3D printing methods has turned out to be a promising solution to these difficulties. Every year, 3D printing becomes a more powerful and indispensable tool in many fields of science [3].

Additive manufacturing (AM), commonly known as 3D printing, is a promising group of techniques for the quick and precise design of various types of products with complex geometries. Additive manufacturing techniques are based on layer-by-layer application of the printed material, resulting in a three-dimensional physical model. Three-dimensional models can be created through various types of imaging techniques or through the use of computer-aided design (CAD) systems. Three-dimensional printing is a highly desirable approach to manufacturing as it enables the creation of designs with complex geometry and architectures that are not possible with conventional manufacturing processes [4,5,6,7]. In addition, this technique is much cheaper and faster. The design flexibility of this process also enables a reduction in human errors during prototyping and the production of more components in one process cycle.

**Figure 1:**
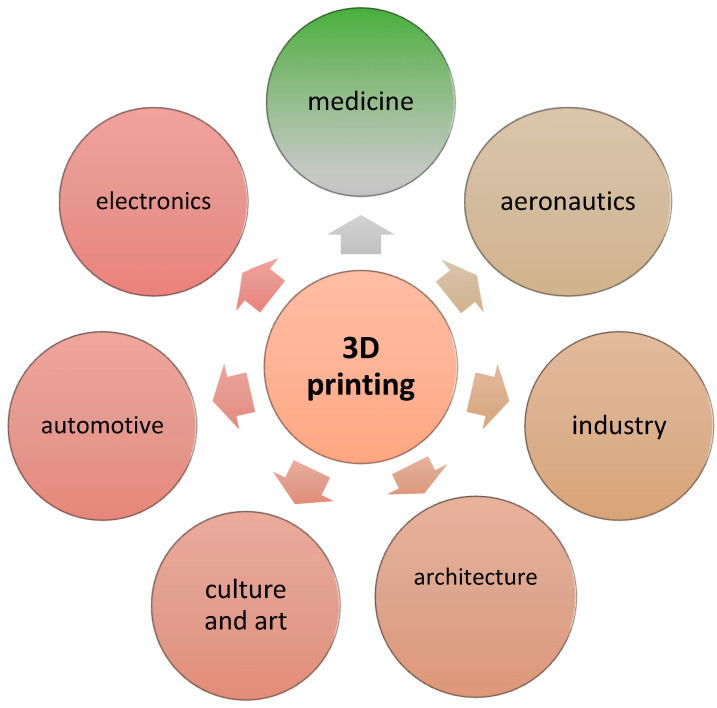
Applications of 3D rapid prototyping technology.

Three-dimensional printing technology has been evolving at a very fast pace since the early 1970s due to the wide application of this technology in every industry and everyday life (Figure 1). Three-dimensional printing methods have recently gained more and more importance in the fields of medical and pharmaceutical applications. The introduction of 3D printing in pharmacy has made it possible to model new multifunctional drug delivery systems that are characterized by an accelerated release process [8]. In terms of the medical aspect, 3D printing is currently widely used for the production of anatomical structures, surgical templates and implants in bone surgery and dentistry. Recently, 3D printing has been applied in such fields of medicine as orthopedics, spine surgery, maxillofacial surgery, neurosurgery and cardiac surgery [9].

3D printing and additive manufacturing consist of a process of building three-dimensional objects from a digital file supplied to the printer software. The geometry of the object is designed using computer-aided design (CAD) software using commercial industry standard programs such as SolidWorks or AutoCAD. On the other hand, the creation of 3D models is usually performed using programs such as Blender, FreeCAD, Meshmixer and SketchUp, which are available in free versions. The designed object is saved in the appropriate file format that can be read by the 3D printer software [10,11,12,13].

An important aspect is the selection of the appropriate biomaterial, which should be characterized by biocompatibility, ease of printing with adjustable degradation rates and the ability to imitate morphologically living tissue. The choice of biomaterial for the 3D printing mechanism depends on the intended use of the final product. For example, a biomaterial characterized by high mechanical stiffness and a longer biodegradation time can be successfully used for orthopedic or dental applications. However, in the case of applications on the skin or in other visceral organs, the biomaterial used should be characterized by high flexibility and a faster rate of degradation. Most biomaterials are used for orthodontic applications because materials such as metals, ceramics, polymers and composites have high rigidity. Soft polymers, e.g., hydrogels, are widely used in cell bioprinting for tissue/organ production.

Three-dimensional technology uses various types of materials, such as metals or ceramics, but the most commonly used group of materials is polymers. Polymers have found wide application in AM technologies due to their ease of processing and low cost. The material possibilities of polymers for 3D printing depend on their molecular structure, which affects their physicochemical properties and processing ability [6]. Currently, in order to improve the properties of polymers used for 3D printing, various types of fillers, e.g., nanoparticles, are introduced into the polymer matrix.

In additive manufacturing, the material is laid out layer by layer in a predetermined path that reflects the shape of the printed object until the complete geometry of the object is obtained. Depending on the 3D printing process, additive manufacturing can be divided into four categories, including extrusion printing (fused deposition modeling (FDM) and bioprinting) [14,15,16,17], material sintering (selective laser sintering (SLS), electron beam manufacturing (EBM), stereolithography (SLA) and continuous liquid interface production (CLIP)) [18,19,20,21], material binding (binder jetting/inkjet and PolyJet) [22] and object lamination (laminated object manufacturing (LOM)).

This paper summarizes the applications of 3D printing in various fields of medicine and introduces various examples of 4D printing. The referenced research discusses the development of materials science and 3D printing technology with uses in medicine and examples in pharmacy.

## 2. A Review of the Literature

### 2.1. FDM Technique

Fused deposition modeling (FDM) is one of the oldest 3D printing techniques and therefore it is the most common and cheapest type of additive manufacturing technology. In this technique, the thermoplastic fiber passes through a heated printhead, where it is heated to about 0.5 °C above the melting point of the material and is laid layer by layer on the printer’s build platform; the process continues until the full geometry of the product is obtained. Fused deposition modeling printers (which are printers equipped with multiple printheads) can use a wide range of materials and can print at high resolutions from many materials simultaneously. The key role in printing the product is the appropriate, optimal selection of individual process parameters, such as the temperature used (of the extruded material and the working table), the number and height of layers and the angle and thickness of the raster. Appropriate selection of 3D printing parameters affects the final performance of the product, including mechanical properties and pore size.

The most commonly used thermoplastic polymer in the FDM process is acrylonitrile butadiene styrene terpolymer. However, materials such as polylactide, polyamide, polycarbonate and polyvinyl alcohol are just some of the other commonly used printing filaments. Lactic acid-based polymers, including polylactide and polycaprolactone, are well known for their biocompatible and biodegradable properties and are therefore widely used in medical and pharmaceutical applications. These polymers are characterized by low melting points, which facilitate the introduction of drugs without causing the polymers to lose their bioactivity due to thermal degradation. Moreover, these polymers are hydrolyzed in vivo and eliminated by excretion [23,24,25,26]. In addition, polylactide is characterized by higher mechanical strength compared with polycaprolactone, which allows it to be used for products that require/work under loads.

In medicine, FDM is used to produce patient-specific medical devices such as implants, prostheses, anatomical models and surgical templates. Often, thermoplastic polymers are doped with various bioactive agents, including antibiotics [27,28], chemotherapeutics [29,30,31], hormones [32,33], nanoparticles [34,35,36,37] and other oral doses [38,39] for personalized medicine. Combinations of materials such as PCL/chitosan [40] and PCL/β-TCP (tricalcium phosphate) [41] are also used in the FDM process to increase the bioactive properties of scaffolds. The use of this technology to create medical models and non-biocompatible materials such as ABS [42] or thermoplastic polyurethane [43] can be useful for perioperative planning and surgical simulations. These models are also used as educational elements for students, and as tools to explain procedures to patients before they undergo surgery.

**Figure 2:**
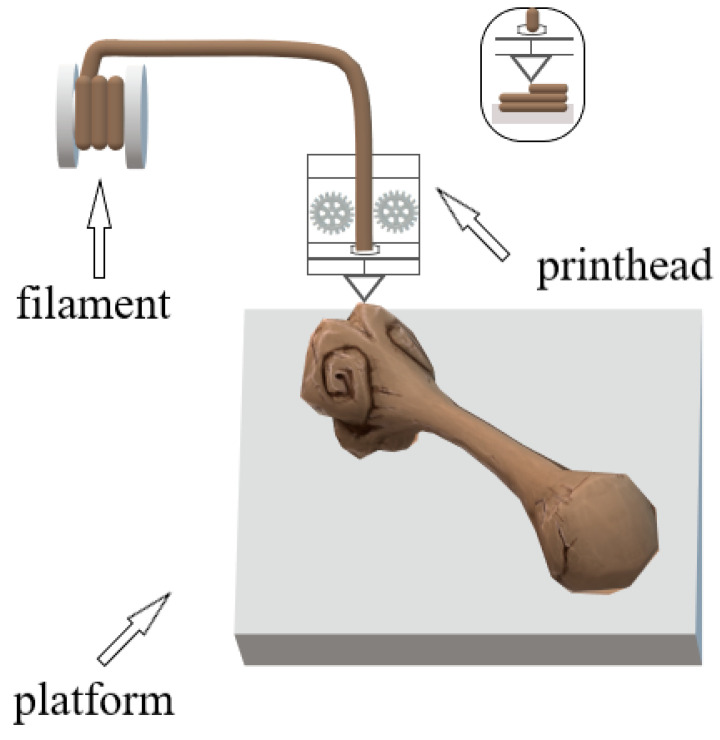
Scheme of operation of a 3D printer in FDM technology.

This technology is based on the selective deposition of molten material layer by layer by using a temperature-controlled printhead, as shown in Figure 2.

Standard filaments used in FDM technology are made of thermoplastic polymers, such as polycarbonate, acrylonitrile butadiene styrene (ABS) or polyamide (PA). However, biopolymers such as PLA, PCL and PLGA are most often used for the production of bone scaffolds. In addition to polymers, this method can also be used to process composites, biocomposites, nanocomposites and fiber-reinforced composites [44,45,46,47,48].

FDM technology ensures the use of a wide range of materials, low cost and short process time. It also controls the pore size and morphology of the products. However, during printing, voids can occur, which adversely affects the properties of the resulting products. Moreover, this technology has a low resolution of about 40 μm. This relatively low-resolution value is related to the difficulties in processing molten thermoplastics through a small-diameter nozzle [8,49,50].

The table below presents a comparison of the printing temperatures and the properties of individual thermoplastics (Table 1).

FDM technology has found application in many industrial fields, including the aerospace, automotive, marine, sports equipment, electrical and medical industries. In the automotive industry, FDM is mainly used in the printing of instruments, handles and prototypes. Most applications in the medical industry are still being tested for the biocompatibility of composite parts printed with FDM [50].

In the extrusion-based bioprinting method, materials, so-called bioinks (biomaterials filled with cells and other biological materials, used for 3D printing), are extruded through the printhead using pneumatic pressure or mechanical force. As in the case of FDM, the materials are laid down continuously, layer by layer, until the required shape of the product is obtained. Because this process does not involve any heating procedures, it is most commonly used to produce engineered tissue structures with cells and growth hormones. This 3D printing process allows small units of cells to be deposited accurately, with minimal cell damage caused by the process. Advantages such as precise cell deposition, control over cell distribution rate and process speed have greatly increased the applications of this technology in the fabrication of living scaffolds.

Currently, research is being conducted on many different polymers for use in bioprinting technology. Commonly used natural polymers include collagen [51], gelatin [52], alginate [53] and hyaluronic acid (HA) [54], and synthetic poly(vinyl alcohol) [55] and polyethylene glycol. Often, bioinks are post-treated via chemical or UV cross-linking to improve their mechanical properties. Depending on the type of polymer used in bioinks, biological tissues and scaffolds of varying complexity can be produced. With this technique, multiple printheads carrying different types of cell lines can be obtained for printing a complex multicell construct. Extrusion bioprinting has been used to fabricate scaffolds for the regeneration of bone [56], cartilage [57], the aortic valve [58], skeletal muscle [59], neurons [60] and other tissues. Despite all this success, material selection and mechanical strength still remain major problems in bioprinting [61,62,63].

In material sintering, the powdered form of the printing material in a tank is fused into a solid object via physical (UV/laser/electron beam) or chemical (binding liquid) sources. Thanks to this technique, objects made of photocurable polymer resins can be 3D printed with high accuracy and resolution. The main limitation of this technology is the small number of materials that can be used for the process. Most currently available photocurable resins are based on low molecular weight polyacrylate or epoxy resins. Composite polymer–ceramic resins, consisting of calcium phosphate salts based on hydroxyapatite, are commonly used for biomedical applications.

The inkjet or binder jet printing process is similar to the previously described material sintering, only instead of melting the powder bed with a laser or electron beam, a binder liquid is selectively dropped onto the powder bed to bond the materials layer by layer. This technique enables the use of two types of printheads, thermal and piezoelectric. Widely used materials include water, phosphoric acid, citric acid, poly(vinyl alcohol) and poly-DL-lactide [64]. A wide range of powdered substances, including polymers and composites, are used for medical applications and tissue engineering. Finished 3D printed objects are often postprocessed to improve their mechanical properties [65,66,67,68,69].

In the PolyJet printing technique, as in the case of inkjet printing, layers of photopolymer resin are sprayed onto the build plate and cured simultaneously with a UV light source. Unlike the inkjet process, multiple types of materials can be injected and cured simultaneously. This gives us the opportunity to create a complex multi-material object. Thanks to these capabilities, PolyJet is widely used in medicine to produce anatomical models for surgery planning and preoperative simulations. Objects with high resolution and varying modular strength can be 3D printed with high dimensional accuracy using the PolyJet technique. Since the UV source is right next to the spray nozzle and cures the resin instantly, post-treatment of the structure is not necessary. Many types of photopolymers such as ABS, Veroclear, Verodent and Fullcure are commercially available for use in PolyJet printing [70,71].

In laminated object manufacturing technology, thin layers of paper, plastic or sheet metal are glued together layer by layer and cut to the required shape using a metal knife or laser. The process is inexpensive, fast and easy to use. It produces relatively lower-resolution objects and is used for multicolor prototyping.

### 2.2. SLA Technology

**Figure 3:**
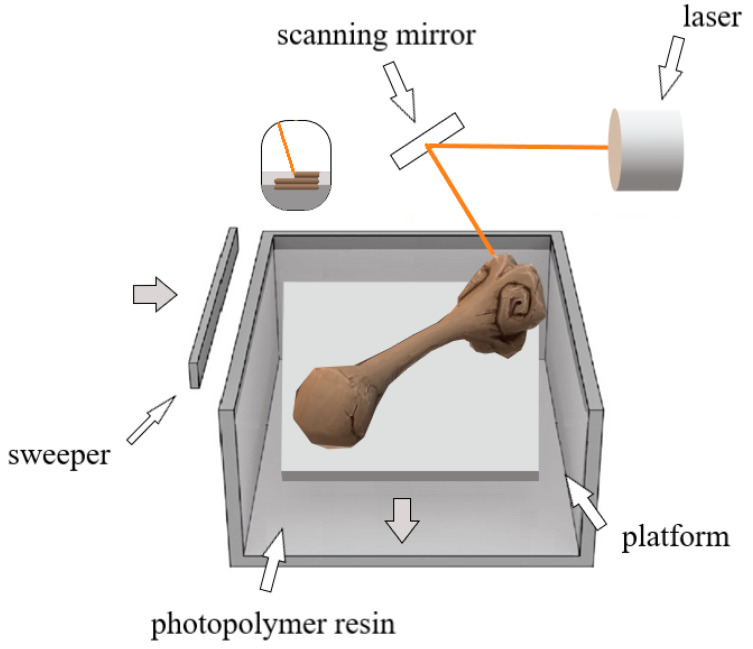
Scheme of operation of a 3D printer with SLA technology.

SLA (stereolithography) technology is the basic method of rapid prototyping, developed in 1984 by Charles Hull [4,47]. This method uses the photopolymerization process. The manufactured object is created as a result of selective hardening of the material with laser light. The schematic process of SLA printing is shown in Figure 3.

A high-energy light source, e.g., ultraviolet (UV) light, and photoreactive resin or monomer solutions are used to produce structures using the SLA method. The monomers used for SLA printing have in their structure acrylic or epoxy groups that can be activated using radiation [47].

This method has been investigated for the creation of various microdevices for medical applications, including tissue scaffolds. By using this method, constructions with high resolutions and precision compared with other 3D printing methods can be obtained. However, this method is expensive, and its use is limited by small material resources and a long process time [6].

SLA uses an ultraviolet (UV) scanning laser to cure layers of liquid photopolymer resin. Each layer is solidified in the xy direction, and the build platform gradually descends in the z direction to be cured. The photopolymerization process merges the layers, which in turn stiffens and strengthens the structure. SLA is a multifunctional and versatile technique. Many resin systems are applied using this method [72].

### 2.3. SLS Technology

**Figure 4:**
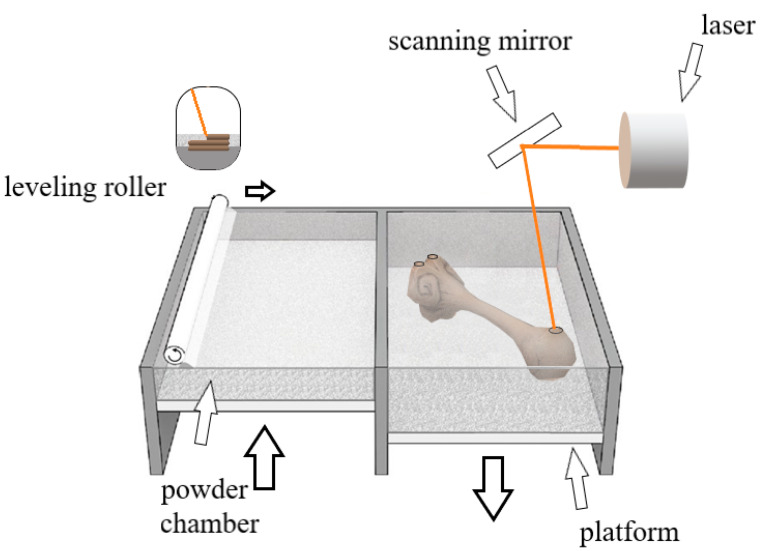
Scheme of operation of a 3D printer with SLS technology.

Another frequently used AM technique is SLS (selective laser sintering) technology. This method is based on the successive melting and sintering of polymers or ceramic granules using a programmed laser beam with layer-by-layer production, as shown in Figure 4 [3,73,74]. The main principle of operation for the SLS technique is that the plastic, which is applied in the form of powder, is selectively plasticized with a laser beam. The material is ultimately applied to the working platform with the use of an appropriate tool. The whole process takes place in strictly defined thermal conditions surrounded by a protective atmosphere. After applying one layer, subsequent layers are applied. The whole process is repeated. As a result of the SLS process, models with complex geometry are created with no need to use support material (the function of support structures is performed by unsintered powder).

SLS technology is widely used in tissue engineering, because regardless of the type of material used, the product is characterized by high resolution and has a structure with numerous and large pores, which enables better cell regeneration. However, too many pores translate into a reduction in the mechanical properties of the products [1]. One of the main advantages of selective laser sintering is that the powder left on the platform acts as a support during the construction of the component. Thanks to this, the process does not require printing a separate support material and enables the production of products with complex geometry [6].

### 2.4. Four-Dimensional Printing Technology

The introduction of 4D printing to industry has revolutionized all sectors of the economy. Current research shows that 4D printing, although still at an early stage of development, is a promising technology that brings huge benefits [75,76].

Research has shown that most materials used for 3D printing, such as metals, plastics and ceramics, are not applicable in 4D printing. Four-dimensional technology uses intelligent or composite materials that change shape under the influence of external stimuli, such as temperature, humidity, light or pH [75,76,77,78]. Materials for 4D printing are classified according to many criteria, most often due to the type of stimulus to which they respond. Currently, researchers devote a large amount of attention to hydrogels, because they are very interesting intelligent materials that react vigorously with water. In addition, these materials can react to the effects of the world and electricity [77,78].

**Figure 5:**
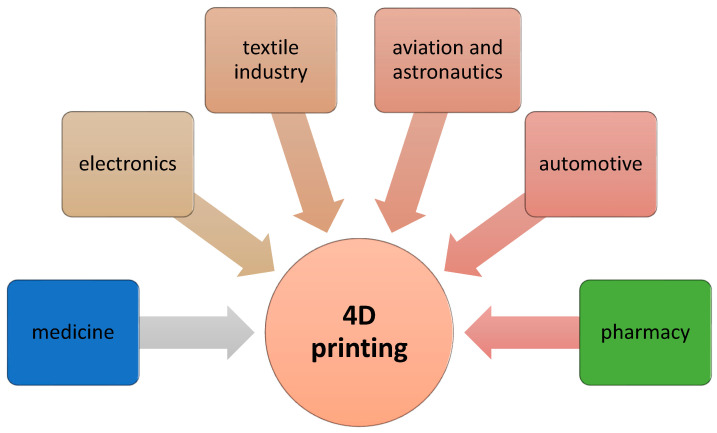
Typical applications of 4D printing in various industries.

Recently, it has been shown that 4D printing technology is a future-proof technology that can be used in every industry (Figure 5). In fields such as medicine, pharmacy or tissue engineering, this technology can produce biocompatible products (stents, artificial organs) that show a change in deformation in a physiological environment [79,80]. Four-dimensional printing can also be used in aviation and construction in order to obtain elements that should have greater strength and flexibility.

### 2.5. Vacuum Casting (VC) Method

**Figure 6:**
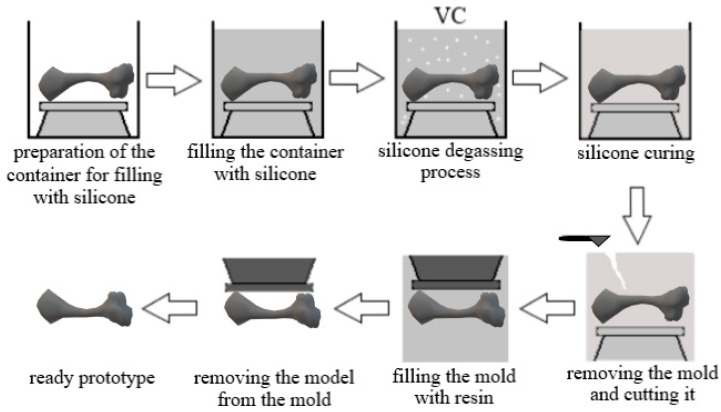
Diagram of the vacuum casting process.

One of the commonly used methods of casting industrial parts is the vacuum casting method. This model is used to duplicate existing parts in small series. This method is one of the most widespread and easiest-to-adapt methods of tooling production. The process can be divided into several stages. The first one is based on the preparation of a silicone rubber mold in order to cast previously computer-designed parts (master pattern). At this stage, the designed prototype is poured with silicone. After the mold is cross-linked, it is divided into two parts and the prototype is removed. The next stage is placing the mold in a vacuum chamber, and its empty part is filled with the designated material composite. The vacuum conditions cause the removal of gas bubbles, resulting in uniformity in the mold and accurate reproduction of the model [79]. In the third step, the resin is cured in the oven and, after cross-linking, is carefully removed from the mold. The scheme of the VC method is shown in Figure 6 [46].

The vacuum casting method in silicone molds uses less expensive tools and materials, and is therefore a good alternative to existing microform production methods. In addition, silicone rubber is characterized by good thermal stability and chemical resistance, which allows one to make castings from a wide range of resins. This material is made of Si-O and Si-C chains in a matrix of linear polymers, which enables the free exchange of molecules and low interfacial energy. Therefore, the cast material is not exposed to a reaction with the surface of the mold [81,82,83,84,85,86,87,88,89,90]. 

In order to obtain subsequent parts through vacuum casting, chemically hardening resins, such as polyester, polyurethane or epoxy resins, are mainly used. Specific functional or aesthetic properties of castings are obtained by mixing resin with fillers. They can be both coloring pigments that give the product aesthetic value, as well as silicas or metal powders that affect the final physicochemical properties of the product [83,91,92]. Currently, there is an increase in interest in hybrid composites, and their appropriate design allows the use of the characteristics of individual components. This results in minimizing the disadvantages resulting from their individual use [93]. Oleksy and others used the vacuum casting method to produce a gear made of a composite containing epoxy resin and hybrid filling. It was found that this hybrid has a regular layered morphology, which increased the mechanical strength of the product. The tensile strength increased by 44% [93,94].

**Figure 7:**
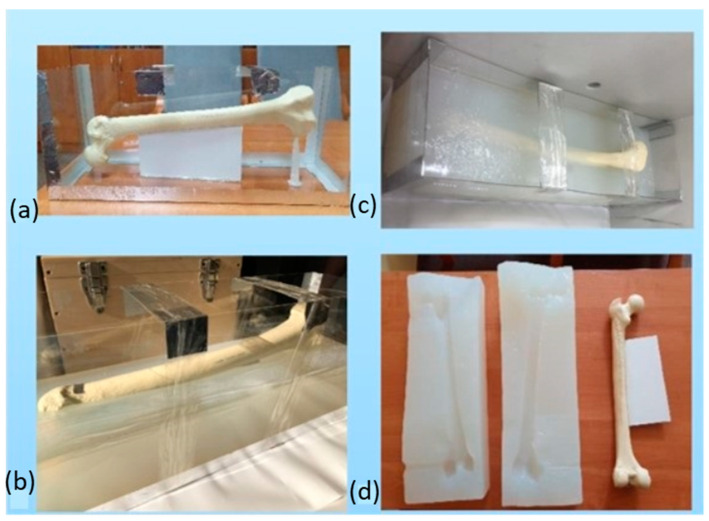
The process of obtaining a mold used in vacuum casting technology: (**a**) the base model with the mold housing; (**b**,**c**) the process of pouring the base model with silicone rubber; (**d**) view of the mold with the model of anatomical structure.

This technology can be used to obtain silicone molds in which, through vacuum casting, we obtain casts of anatomical structures often used by orthopedic surgeons for preoperative training. Figure 7 shows the process of obtaining a silicone mold.

The first step is to form a 3D model with a given and acceptable form. An essential requirement is that the objects intended for casting must be made in accordance with the principles of injection molding. For this purpose, appropriately dedicated software is used: AutoCAD, SolidWorks or CATIA. The designed 3D model is a sketch that matches the quality of the master model. After the pattern is developed, the mold is cast. The master model, complete with casting cores, inserts and gates, is suspended in the casting box. The formed road is placed in a vacuum casting box, and liquid silicone is poured around it, filling in all the details. It is then placed at 40 °C for several hours to cure. The box and dividers come out after the silicone dries and sets. To finish, the cavity of the negative mold of the piece is exposed by gently splitting the mold with a knife. The mold is then put back in place and the sprue gates are attached to the mixing and pouring vessel. Vacuum casting resins and color pigments are thoroughly mixed and vented under vacuum during the auto-potting process. Then, a vacuum is created in the mold and resin is poured inside. After pouring the resin into the mold, it is heated in a drying room until it hardens. The casting can be removed from the mold when it has hardened. Once casting is complete, the gate and risers can be removed and final finishing touches can be applied.

## 3. Discussion

Three-dimensional printing is defined as the most modern technologies that enable and support the precise and quick production of objects of various structures, forms and materials. The latest advances in 3D printing technology and in various fields (from medicine to industry) enable doctors and engineers to design and print various types of templates, implants and structures [95]. Also, in the pharmaceutical department, 3D printing is used because it offers significant advantages compared with traditional pharmaceutical processes. Advances in 3D printing technology may lead to the design of a suitable 3D printing device capable of producing formulations with the intended drug-release abilities. The methods under development appear to be transformative tools with greater flexibility in pharmaceutical production. Three-dimensional printing technology is a process that enables the production of three-dimensional preparations under digital control. Such a process can provide developed regimens for the treatment of patients with various ailments [96]. Major 3D printing technology platforms in the medical field include inkjet printing, binder jetting, fused fiber fabrication, selective laser sintering, stereolithography and pressure-assisted microsyringes [97]. Another area of research that 3D printing is expected to revolutionize is the production of implantable bioresorbable drug-release scaffolds (stents). The ability to customize and create customized, tailor-made bioresorbable scaffolds has the potential to address many stent-related challenges, such as inadequate stent size and design, to abolish late stent thrombosis, and to aid in arterial growth. Three-dimensional printing offers rapid prototyping and an efficient method of producing stents, allowing one to customize designs to individual needs [98].

The medical field is more willing and more likely to utilize the possibility of rapid production for anatomical models; the most common is printing with plasticized plastic—FDM (fused deposition modeling). The universality of this method results from the wide availability of low-cost devices and the wide range of materials used in this technology. In addition, the technology is developed dynamically, which results in improved accuracy of the reproduction of manufactured models while maintaining a low price and short printing time. FDM technology is used in the case of illustrative models that show the relationships of structures in the modeled organ, enabling improvements in preoperative preparation; they are also a didactic element for young doctors, students and patients themselves. In the case of simulating operations or creating a complex pathology that requires high accuracy of elements, models made with FDM technology do not fully fulfill their role. Therefore, when producing phantoms, other technologies are used, e.g., stereolithography technologies such as SLA or PolyJet. Their greatest advantages are precise mapping of the shapes, high accuracy of the manufactured elements and lower layer thickness values. Models printed with these technologies are more detailed, which is why in the case of highly complex operations, e.g., cardiac surgery, where it is also necessary to model the network of blood vessels, these methods are used. It should also be mentioned that due to the way SLA and PolyJet printers work, we have the opportunity to use resins and other materials with reduced hardness, which translates into the possibility of producing models imitating living soft tissues.

SLA as a 3D printing technique is very fast and very accurate. It allows one to obtain ready-made molds of uniform quality. Molds can be produced using SLA on the principle of photopolymerization. Various 3D printing technologies have been introduced. SLA (as one of the available methods) has commercial applications. SLA has several advantages over other methods, such as cost-effectiveness, controlled integrity of materials and faster speed. The development of SLA enabled the development of printed pharmaceutical devices. Given current trends, it is expected that SLA will be used in parallel with conventional 3D model fabrication methods. This 3D printing technology can be used as a novel drug delivery tool [21].

Thanks to this, printed models can be used to simulate operations, even many times, as is the case in IPCZD. These technologies are more expensive and time-consuming than the FDM method. The decision on the selection of technology is related to the specific case, the time and budget available to the hospital and the requirements to be met by the model [90,99,100].

The production of implants or prostheses is dominated by the technologies of selective laser sintering of metal powders—DMLS (direct metal laser sintering)—using titanium powders which are also used in the classic production of endoprostheses.

Due to the high loads occurring in the areas of application of endoprostheses or some titanium plates, they are impossible to print with other technologies based on production from plastics [100,101].

Recently, 3D printing has been applied more and more in the field of biomedicine, including the clinical application [102] of lumbar spine [103] and long bone prosthetics in orthopedics [104] and the clinical application of oral jaws [105] and skulls for neurosurgery [106]. In addition, tissues and organs including skin [107], blood vessels [108], and hearts [109], etc. have all been produced in large quantities via this technique. 

Material sciences are advancing rapidly, and they are closer than ever to engineered tissue models capable of predicting preclinical responses to therapies, modeling disease and being used as lifesavers of cardiac function after native myocardial injury.

However, the main problem of 3D printing is in vivo integration. In this review, we highlight the seminal and recently published work that has influenced and pushed the field of tissue bioengineering towards achieving tissue vascularization [110,111]. Three-dimensional printing shows great promise in its ability to pattern hierarchal architectures of the heart and its vasculature, but is ultimately hindered by the material properties of bioinks and the resolution of the print when printing connective capillary beds. In cases of irreversible damage, treatment is difficult due to the inherent complexity of the gradient nature of biochemical and mechanical properties and the microscale composition of many cell types.

Three-dimensional printing has emerged to fabricate patient-specific bioactive scaffolds that possess controlled microarchitectures for bridging bone defects in complex configurations [112]. 

Tissue engineering applications of 3D bioprinting, in particular, have attracted the attention of many researchers. Three-dimensional scaffolds produced via the three-dimensional bioprinting of biomaterials (bioinks) enable the regeneration and restoration of various tissues and organs. These 3D bioprinting techniques are useful for fabricating scaffolds for biomedical and regenerative medicine and tissue engineering applications, permitting rapid manufacture with high precision and control over size, porosity and shape [113].

Three-dimensional printing is a rapid prototyping technology which assembles biomaterials, including cells and bioactive agents, under the control of a computer-aided design model in a layer-by-layer fashion. It has great potential in organ manufacturing areas with the combination of biology, polymers, chemistry, engineering, medicine and mechanics. At present, 3D printing technologies can be used to successfully print living tissues and organs, including blood vessels, skin, bones, cartilage, kidneys, the heart and the liver. The unique advantages of 3D printing technologies for organ manufacturing have improved the traditional medical level significantly. In this article, we summarize the latest research progress on polymers in bioartificial organ 3D printing areas. The important characteristics of the printable polymers and the typical 3D bioprinting technologies for several complex bioartificial organs, such as the heart, liver, nerves and skin, are introduced [114].

Tissue engineering has been recognized as a highly promising strategy to solve the problems of organ donor shortage through the fabrication of artificial organs/tissue. This includes the prospective technology of 3D printing, which has been adapted to various cell types and biomaterials to replicate the heterogeneity of urological organs for the investigation of organ transplantation and disease progression. The literature shows that advances in this field towards the development of functional urological organs or disease models have progressively increased. Although numerous challenges still need to be tackled, 3D printing has the potential to fabricate functional urological organs for clinical transplantation and in vitro disease models [115].

The book by Douglas describes the challenges and accomplishments in the bioprinting of blood vessels, cartilage, skin, bone, skeletal muscle, neuromuscular junctions, liver, heart, lungs, kidneys and so-called organs-on-a-chip, as well as the challenges of providing a blood supply and nerves to bioprinted tissues [116].

Three-dimensional printing approaches in medicine and pharmacy provide a solution by embedding tissue-specific cues, biomaterials and cell types at high resolution in a hierarchical and complex architecture, offering promising prospects for regenerating tissue interfaces.

Each tissue interface has specific characteristics that require the development of new bioinks to achieve their complexity. The biochemical composition of bioinks can be fine-tuned by adding growth factors, bioactive molecules, molecular signals or varying numbers of cells and cell types and arrays to achieve the natural properties of tissue interfaces, as well as help better integrate with the host tissue. The efficiency of the printing process depends on the properties of the polymer or bioink. The developed bioink should meet the requirements for printability, including viscoelasticity, gel time and cross-linking mechanism. In addition, printing systems should also allow custom printing patterns to allow the microscale material to be deposited in the appropriate and desired geometry. Each approach to bioprinting has advantages and disadvantages regarding bioink characteristics, printability and precision. While some can generate constructs at nanoscale resolution, the final structure may not provide a suitable microenvironment for cell viability and functionality or meet the target physical properties of the tissue interface [116,117].

Modification of physical structures’ properties, such as pore size, fiber diameter and bioink types, enabled the development of specific tissue analogues with different functions in different tissue zones.

Also crucial is the postprocessing step that determines the functionality of the biofabricated structure, including viability, proliferation, cell differentiation and remodeling of the construct. While biofabrication technologies facilitate the controlled deployment and realization of the physicochemical and geometrical properties of tissue interfaces, traditional cell culture methods need to be modified to enable multicell coculture systems. An imbalance between the proliferation and differentiation of different cell types can hinder the successful maturation of tissue interfaces in vitro.

Initial steps have been made in the development of interface tissue models for drug discovery or small organ models. We anticipate that their clinical applications will soon emerge. Due to potential issues with ethical regulations and social barriers, animal models are most often used to predict the body’s response to engineered, viable biomimetic tissue interface structures, but they may not be biologically and mechanically biocompatible with the human body. Since tissue interfaces are mechanically unstable, disintegration of the implant and failure to integrate with the host tissue are highly likely. In addition, the passage of clinical infections into host tissue can cause serious problems. Ongoing development, however, can help overcome transplant challenges and facilitate rapid integration.

Three-dimensional printing, with its personalized and highly customized characteristics, has great potential in the pharmaceutical industry [117]. 

We found that 3D printing technology has the following applications in pharmacy:

- To print pills according to the individual condition of the patient [118];
- To make the dosage more suitable for each patient’s own physical condition [119,120,121,122];
- To print tablets with specific shapes and structures to control the release rate [123,124];
- To precisely control the distribution within cells [125];
- To develop biomaterials to build organs [126,127,128,129,130,131,132,133,134,135,136,137,138,139,140,141,142,143,144,145,146,147,148,149,150,151];
- To develop biomaterials to build organs-on-a-chip for drug testing [152];
- To make transdermal microneedle patches to reduce pain in patients [153].

Three-dimensional bioprinting is used to produce personalized and complex products on demand that increase the availability, effectiveness and safety of drug therapies and delivery systems. In addition, this review describes the ability of 3D bioprinting to produce patient-specific tissues and living cell systems (e.g., organ-on-a-chip constructions).

Four-dimensional printing (printing materials designed to change over time or under stimuli) can be used to overcome many of the inherent limitations of conventional three-dimensional printing technologies. It also provides a comprehensive insight into the critical future of bioprinting; the key requirements for 4D printing include material programmability, multi-material printing methods and precision designs for meticulous transformations and even clinical applications [154,155,156,157].

However, bioink formulation is one of the main challenges in the 3D bioprinting of cell-laden scaffolds for human tissues. Bioink consists of a biomaterial solution (ink) and cells in the presence or absence of growth factors [158]. 

Some bioprinting companies also provide professional commercial software (e.g., Axway TradeSync Integration Manager^®^ version 4, BioAssemblyBot^®^ 400 and BioCAD^®^ version 0.0.2.2) to design, draw and print multiscale structures ranging from cells to tissue constructs. Three-dimensional bioprinting is an emerging technology expected to revolutionize the fields of tissue engineering and regenerative medicine. The conventional tissue engineering approaches use three-dimensional (3D) prefabricated scaffolds as matrices to load cells [158].

## 4. Materials and Methods

The types of publications considered for analysis mainly included review and articles in the English language. A search focused on 3D printing was conducted on PubMed, Google Scholar, Elsevier and Scopus from inception to June 2023. The search terms included the phrases “3D printing” and “3D technology”. The authors of this review worked on the basis of an agreed scheme, selecting articles based on their title, language, abstract and access. Duplicate records were removed.

## 5. Conclusions

The use of 3D printing in medicine continues to grow due to its capabilities, such as personalization of medicine, cost-effectiveness, speed and increased productivity. Three-dimensional printing is rapidly gaining wider use in health care and pharmacy. The technology has shown success in improving surgical techniques through the development of tissues, organ models and a new model of drugs and drug delivery. 

The ability to use multiple materials and colors, as well as reduced material waste due to higher deposition accuracy, are two major advantages of this process that are driving its demand. Three-dimensional printing could also be used to monitor smart implant performance.

Four-dimensional printing can be used to overcome many of the inherent limitations of conventional three-dimensional printing technologies. It also provides a comprehensive insight into the critical future of bioprinting. Four-dimensional printing provides advantages including material programmability, multi-material printing methods and precision designs for meticulous transformations and even clinical applications. Additive manufacturing has revolutionized the field of medicine and continues to grow rapidly. Popular clinical applications include the manufacture of implants and prostheses tailored to the needs of patients; engineering scaffolds for regenerating biosynthetic tissues and organs; personalization of drug delivery systems; and anatomical modeling for perioperative simulations.

## Figures and Tables

**Table 1:** Summary of printing temperatures and properties of various thermoplastic materials used in 3D FDM printing.

Thermoplastic Polymer	Acrylonitrile Butadiene Styrene (ABS)	Polylactidic Acid (PLA)	Polyetheretherketone (PEEK)	Polyetherimide (PEI)	Polycarbonates	Acrylonitrile Butadiene Styrene (ABS)	Polyamide (Nylon)
Printing temperatures (°C)	220–250	190–220	350–400	355–390	150	210–270	230–260
Properties	High strength, flexibility and durability	Biodegradable, brittle	High mechanical strength, durability and flexibility	High specific strength, fire resistance and chemical resistance	Thermoplastic, strong, and some grades are optically transparent	High rigidity, good weldability and insulating properties	High strength, elastic

## Data Availability

All data have been included.
